# When your eye patient is a child

**Published:** 2010-03

**Authors:** Arvind Chandna, Clare Gilbert

**Affiliations:** Consultant Paediatric Ophthalmologist and Chair: Vision for Children, Alder Hey Children's Hospital. Liverpool, UK.; Co-director, International Centre for Eye Health, London School of Hygiene and Tropical Medicine; Clinical Advisor, Sightsavers, UK.

**Figure:**
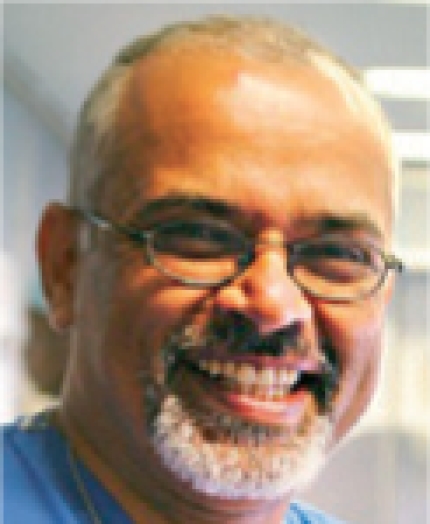


**Figure:**
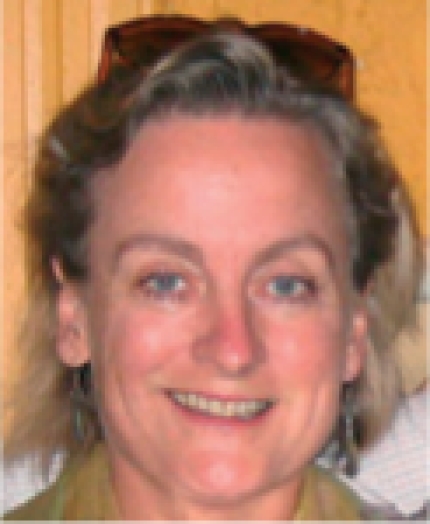


In this issue of the journal, we address the eye health needs of young children, focusing on those aged less than six years old. All of you who have tried to examine or measure the visual acuity of young children will know that this can be very challenging and difficult; it can be very tempting to give up and send the child home, particularly if the clinic is busy. We hope that, after reading this edition of the journal and putting into practice some of the practical suggestions, you will feel more confident in managing young children. Also, if referral is needed, you will have a better idea of the degree of urgency required and how this should be communicated to parents.

**Figure:**
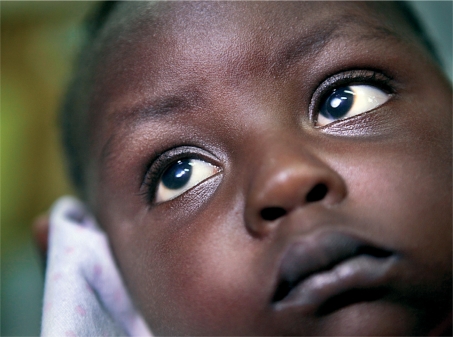
A young child with bilateral cataract. TANZANIA

CCBRT/Dieter Telemans

## The impact of eye disease in children

It is said that almost three quarters of a child's early learning comes through vision and that over one third of the adult visual cortex responds to visual stimuli. This tells us that vision is not only very important for early development in infancy, but also that visual information is used and processed by many different parts of the brain. It is, therefore, not surprising that visual loss early in a child's life can have a major Impact on that child's development, leading to delays in crawling and walking, for example. Preventing visual loss, or ensuring that a child has the correct treatment at the correct time to restore visual function, will have a major impact on the child's development.

The impact of visual loss also extends beyond the child to the family: studies have shown that having a disabled child can increase stress and depression among parents and can lead to increases in divorce or separation.^1^ Conversely, some families find that it brings them closer together, as they jointly share the challenges of living with a disabled child.

## Listening to the mother

All mothers, regardless of educational attainment, want what is best for their child and also know their child very well. They observe their children closely over long periods of time and under different lighting conditions and circumstances. Mothers will notice if there is something wrong with the eye or eyes, or if their child is behaving differently. One of the key messages of this issue is that health workers need to listen to, and believe, mothers: they know more about their child than anyone.

With this in mind, the article on page 4 is based around what parents might say when they bring their child to see you. We hope that you will find this approach useful.

## The challenges

Assessing visual acuity in young children can be very difficult indeed, but children aged five years or above can usually be tested using a Snellen E or Landolt C chart. (**Tip:** If children can reach over the top of their head with one arm, and touch the opposite ear, then they are at least five years old.) Below the age of five years, other methods need to be used to assess vision, such as matching tests, or recognising or finding small objects against a plain background. However, even in tertiary referral hospitals where all the latest equipment and acuity testing charts are available, formal visual acuity measurement is not always possible. Once again, we have to rely on what the mother has noticed or on what we can detect about the child's visual behaviour. The chart on page 5 tells you what a normally sighted child should be able to do at different ages, so allowing you to identify children whose vision may be a cause for concern.

Examining young children can also be challenging as they do not understand what you are doing and are likely to be frightened of the whole experience. This issue contains some tips on how to examine babies and young children (page 6). There are also practical suggestions on how to look after a young child in hospital and how to support parents (page 16) as well as how to make a facility child friendly (page 18). There is also an article on understanding, diagnosing, and managing strabismus/squint (page 12).

## You can make a difference

It is important to realise that the correct action taken by an eye care worker can play a major role in preserving the sight of a child; correct action can even save a child's life! For example, identification and correct referral of a child with a white reflex, which may indicate retinoblastoma, may lead to life-saving treatment - retinoblastoma is often fatal if not treated early. Correctly diagnosing the signs of vitamin A deficiency and giving a child high-dose vitamin A will also reduce the risk that the child will die. Studies undertaken in Indonesia indicate that children with Bitot's spots and night blindness are fifteen times more likely to die than children without these signs.

Congenital or developmental cataract, one of the commonest treatable causes of blindness in children, requires early detection and treatment to prevent permanent visual impairment from amblyopia (‘lazy eye’). This is also likely to be noticed by the mother first. Health workers can check for cataract using the red reflex test (page 11), which should ideally form part of the routine examination of all newborn babies.

Even when you suspect a child with an eye abnormality will not benefit from medical treatment, you should still refer that child to an ophthalmologist or tertiary eye care centre. Such children may be helped by refraction and low vision services, and it is important to refer them as soon as possible to minimise the impact of any visual impairment on their development.

## Beyond the clinic

We encourage you to think about what you can do beyond the clinic to reduce eye disease and visual loss in children. For example, you could talk to staff who work in maternal and child health clinics, or immunisation staff, and suggest that they refer any child to you if they have concerns. Indeed, a programme in Uganda very successfully trained immunisation staff to look at the eyes of the infants and children they were immunising and ask the mothers whether they had any concerns. This intervention led to many children being referred earlier than they would have been for examination, assessment, and treatment.

You can encourage mothers of young children to ensure they receive all their immunisations and take their vitamin A supplement. Discussions with traditional birth attendants could include the importance of cleaning the eyelids immediately after the head is delivered to prevent conjunctivitis of the newborn (ophthalmia neonatorum).

If you see one child with trachoma, vitamin A deficiency, or measles infection, it is likely that there are other children in the same area who have the same problem. You should find out which community or area the child is from and inform the relevant authority.

**Ten key activities for primary health care workers**

1. Clean the eyes immediately after birth and instil topical antibiotic eye ointment or topical antiseptic eye drops.
2. Give the mother 200,000 international units (IU) of vitamin A immediately after delivery.
3. Promote breastfeeding and good nutrition.
4. Immunise children against measles at nine months and give vitamin A 100,000 IU. Encourage second immunisation for extra protection.
5. Give any child with measles or suspected undernutrition vitamin A 100,000 IU (if less than twelve months) or 200,000 IU (if twelve months of age or older).
6. Keep children's faces clean.
7. Refer any child who cannot see well to an eye care worker as soon as possible.
8. Urgently refer any child with a white pupil or other obvious abnormality to an eye care worker.
9. Refer any child with a serious eye injury or a red eye to an eye care worker immediately.
10. Do not put traditional medicines in the eyes.

**Figure:**
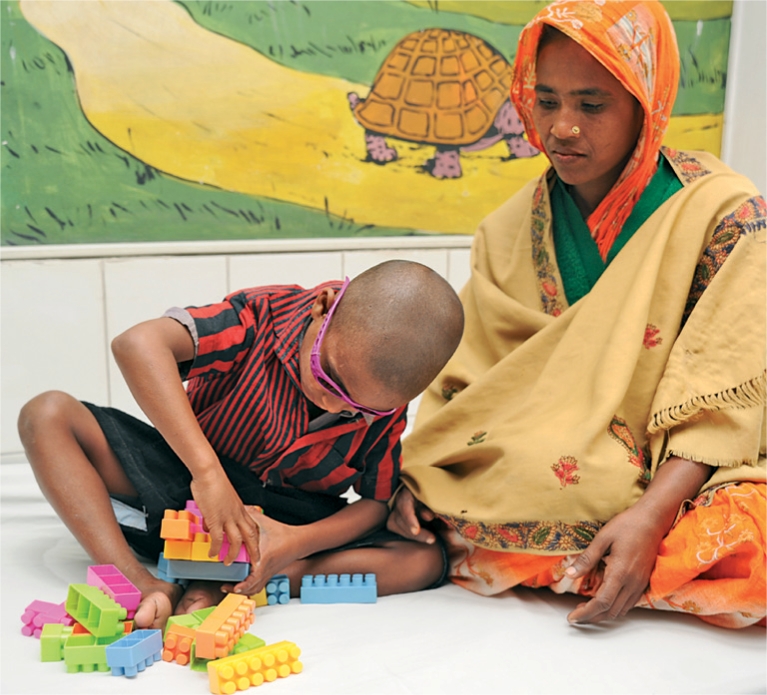
A boy plays after eye surgery. BANGLADESH

Zul Mukhida/Sightsavers

The ten key activities listed in on this page are those suggested by the World Health Organization and are intended for primary level staff.^2^ If these activities were to be widely and consistently implemented, then the effects will spread far beyond eye health and promote the overall health of children.

Health promotion and education, particularly of mothers and women of child-bearing age, is also important. Tell them what they can do to prevent visual loss and eye diseases in their child. The ten key activities are a good place to start.

It is also important to know that most communities hold beliefs about the causes and treatment of illness, including eye conditions. Many communities believe that visual loss of early onset, particularly if it is congenital (i.e., since birth), cannot be treated. As a result, parents do not seek help. Therefore, if a parent attends a health facility very late, do not blame them, as it is likely that they hold the same beliefs as many others in their community.

## In summary

Remember that children are not smaller versions of adults, nor are their eyes smaller versions of adult eyes - children's eye care needs are different, and often more urgent than those of adults. It is true that managing young children in the clinic can be a challenge. However, there is a lot that you and your colleagues, as well as parents and the community, can do to prevent eye disease and visual loss in children. We hope that this issue will inspire you to provide the best possible eye care to children in a way that supports children and parents and makes the experience of the clinic less traumatic for them.

**How many children are blind?**

Under-5 mortality rates (U5MRs) can be used to estimate the prevalence of blindness in children (see Table 1). The justification for this is that many of the conditions that cause blindness in children are also causes of child mortality, such as measles, vitamin A deficiency disorders, meningitis, and congenital rubella.^3^ At the launch of VISION 2020 in 1999, there were estimated to be 1.4 million blind children in the world, almost three quarters of whom lived in low- and middle-income countries.^4^

**Table 1:** Under-5 mortality rates and blindness prevalence estimates in children

**under-5 mortality rate per 1,000 live births**	**Estimated prevalence of blindness per 1,000 children**
0–19	0.3
20–39	0.4
40–59	0.5
60–79	0.6
80–99	0.7
100–119	0.8
120–139	0.9
140–159	1.0
160–179	1.1
180–199	1.2
200–219	1.3
220–239	1.4
240+	1.5

Worldwide, over the last ten years, the total number of children aged 0–15 years has increased slightly, to almost 1.9 billion. However, U5MRs, and hence blindness prevalence estimates, have declined in most countries since 1980.^5^

By multiplying the number of children by the relevant blindness prevalence estimate, it has been possible to calculate that that the number of children who are blind globally has declined by around 10 per cent over the last ten years, to 1.26 million in 2010 (Table 2). Better measles immunisation and vitamin A supplementation are two important public health interventions which have undoubtedly contributed.

**Table 2:** Estimates of the number of blind children worldwide in 2010

	**2010 estimate**		**% change between 1999 and 2010**	
	**Child pop (millions)**	**Blind children**	**In child pop**	**In estimates of blind children**
**Lower in 2010 than in 1999**				
China	340	116,000	0%	−44.8%
Other Asia and Islands	266	136,000	−2.3%	−38.2%
EME + FSE	244	70,000	−1.6%	−22.2%
Latin America and Caribbean	170	71,000	0%	−29.0%
**Not much change**				
Middle East Crescent	241	168,000	+0.4%	−11.6%
India	345	280,000	−1.4%	+3.7%
**Higher in 2010 than in 1999**				
Sub-Saharan Africa	274	419,000	+5.4%	+30.9%
**TOTAL**:	1,880	1,260,000	+0.6%	−10%

EME = established market economies; FSE = former socialist economies. These regions have been combined as some countries were re-designated between 1999 and 2010.

In Table 2, countries are grouped by World Bank region rather than geographical region, because socio-economic factors play such an important role in determining the prevalence and causes of visual loss in children.

The revised 2010 estimate suggests that the greatest changes have occurred in China and the Other Asia and Islands region (which includes Indonesia, the Philippines, and Bangladesh), where U5MRs, and hence blindness prevalence estimates, have dropped considerably and the child population has stayed relatively stable.

In Sub-Saharan Africa, the number of blind children has increased by 31%, in part because this is the only region with a significant increase in the child population. However, in many countries in this region, U5MRs have increased, mainly as a consequence of the HIV epidemic and the large number of children who are orphaned, leading to poorer child health.

**How to estimate the number of children who are blind**

Find the relevant U5MR for your country or region. This can be found in several publications and documents, including UNICEF's State of the World's Children, which is published every year.

To estimate the number of children who are currently blind, use the U5MR of five years ago, i.e. for 2005, as this will reflect the most relevant time period.

Find the U5MR in Table 1 and read off the blindness prevalence estimate.

Multiply the prevalence by the number of children aged 0–15 years in your country to estimate the number of children who are blind.

**Example:** Country P has a total population of 12 million in 2010, and 40% are children aged 0–15 years. The number of children is therefore 12,000,000 × 40/100 = 4,800,000. The U5MR in 2005 was 112 per 1,000 live births. So the prevalence estimate is 0.8 per 1,000 children (from Table 1). In country P there will be 4,800,000 × 0.8/1000 = 3,840 children who are blind.

**Note:** These estimates do not include blindness from refractive error as there is very little data on which to base estimates.
